# Detection measures for visual inspection of X-ray images of passenger baggage

**DOI:** 10.3758/s13414-018-01654-8

**Published:** 2019-01-25

**Authors:** Yanik Sterchi, Nicole Hättenschwiler, Adrian Schwaninger

**Affiliations:** University of Applied Sciences and Arts Northwestern Switzerland, School of Applied Psychology, Institute Humans in Complex Systems, Riggenbachstrasse 16, CH-4600 Olten, Switzerland

**Keywords:** X-ray image inspection, Visual search, Signal detection theory, Detection measures

## Abstract

In visual inspection tasks, such as airport security and medical screening, researchers often use the detection measures *d'* or *A'* to analyze detection performance independent of response tendency. However, recent studies that manipulated the frequency of targets (target prevalence) indicate that *d*_*a*_ with a slope parameter of 0.6 is more valid for such tasks than *d'* or *A'*. We investigated the validity of detection measures (*d'*, *A'*, and *d*_*a*_) using two experiments. In the first experiment, 31 security officers completed a simulated X-ray baggage inspection task while response tendency was manipulated directly through instruction. The participants knew half of the prohibited items used in the study from training, whereas the other half were novel, thereby establishing two levels of task difficulty. The results demonstrated that for both levels, *d'* and *A'* decreased when the criterion became more liberal, whereas *d*_*a*_ with a slope parameter of 0.6 remained constant. Eye-tracking data indicated that manipulating response tendency affected the decision component of the inspection task rather than search errors. In the second experiment, 124 security officers completed another simulated X-ray baggage inspection task. Receiver operating characteristic (ROC) curves based on confidence ratings provided further support for *d*_*a*_, and the estimated slope parameter was 0.5. Consistent with previous findings, our results imply that *d'* and *A'* are not valid measures of detection performance in X-ray image inspection. We recommend always calculating *d*_*a*_ with a slope parameter of 0.5 in addition to *d'* to avoid potentially wrong conclusions if ROC curves are not available.

## Introduction

X-ray baggage screening at airports is an essential component for securing air transportation. To prevent passengers from bringing potential threats onto an aircraft, airport security officers visually search X-ray images of passenger bags and decide within seconds whether a bag contains a prohibited item or is harmless. This task can be described as visual inspection consisting of visual search and decision making (Koller, Drury, & Schwaninger, 2009; Wales, Anderson, Jones, Schwaninger, & Horne, 2009) in line with the two-component model of Spitz and Drury (1978). An airport security officer's (screener's) decision on whether a bag is harmless (*target absent*) or might contain a prohibited item (*target present*) determines whether a secondary search must be conducted at airport security checkpoints (typically using explosive trace detection and a manual search of passenger bags; Sterchi & Schwaninger, 2015). Table 1 presents the four possible decision outcomes and associated terminology from visual search studies (e.g., Biggs & Mitroff, 2015; Eckstein, 2011; Wolfe, 2007, p. 99), signal detection theory (SDT; e.g., Gescheider, 1997, p. 106; Green & Swets, 1966, p. 34), and X-ray baggage screening (e.g., Cooke & Winner, 2007; Schwaninger, Hardmeier, & Hofer, 2005).

**Table 1:** Outcome of decisions depending on stimulus using the terminology of visual search, signal detection theory, and X-ray baggage inspection

	Decision	
Stimulus	Target absent No signal Bag is harmless	Target present Signal Bag requires secondary search
Target absent Noise No prohibited item present	Correct rejection	False alarm
Target present Signal plus noise Prohibited item present	Miss	Hit

*Note. Target present* and *target absent* are terms used in visual search studies (Biggs & Mitroff, 2015; Eckstein, 2011; Wolfe, 2007, p. 99). *Noise, no signal, signal plus noise, signal, hit, miss, false alarm,* and *correct rejection* are terms used in signal detection theory (Gescheider, 1997, p. 106; Green & Swets, 1966, p. 34). The other terms have been used in X-ray baggage inspection studies (Cooke & Winner, 2007; Schwaninger, Hardmeier, & Hofer, 2004)

In detection theory (Macmillan & Creelman, 2005), the percentage of bags that contain a prohibited item that are correctly classified as such is called the *hit rate* (*HR*), whereas the percentage of harmless bags that are falsely considered to contain a prohibited item is the *false alarm rate* (*FAR*). There is a trade-off between the HR and the FAR: If, for example, someone's tendency to respond with *target present* increases, both the HR and FAR will increase. At its extremes, someone could decide to always respond with *target present*, thereby resulting in a HR and FAR of 100%. Individuals with the same ability to detect prohibited items can have different HRs and FARs because of differences in their response tendency (also referred to as *response bias*; Macmillan & Creelman, 2005). SDT provides measures (such as *d'* and *A'*) for assessing detection performance. These can be calculated from HR and FAR and are assumed to be (relatively) independent of the observer’s response tendency (Macmillan & Creelman, 2005, p. 39). Since 9/11, a growing body of research on X-ray image inspection of passenger bags has led to an increasing use of *d'* and *A'* in this domain (e.g., Brunstein & Gonzalez, 2011; Halbherr, Schwaninger, Budgell, & Wales, 2013; Ishibashi, Kita, & Wolfe, 2012; Madhavan, Gonzalez, & Lacson, 2007; Mendes, Schwaninger, & Michel, 2013; Menneer, Donnelly, Godwin, & Cave, 2010; Rusconi, Ferri, Viding, & Mitchener-Nissen, 2015; Schwaninger, Hardmeier, Riegelnig, & Martin, 2010; Yu & Wu, 2015). Moreover, *d'* and *A'* are also frequently used in related domains, such as the inspection of medical X-ray images (e.g., Chen & Howe, 2016; Evans, Tambouret, Evered, Wilbur, & Wolfe, 2011; Evered, Walker, Watt, & Perham, 2014; Nakashima et al., 2015) and visual search tasks with artificial stimuli (e.g., Appelbaum, Cain, Darling, & Mitroff, 2013; Huang & Pashler, 2005; Ishibashi & Kita, 2014; Miyazaki, 2015; Russell & Kunar, 2012).

However, as will be discussed in more detail below, the results of several studies in recent years cast doubt on the validity of using *d'* or *A'* for X-ray image inspection tasks (i.e., visual search and decision tasks). Before discussing these findings, we shall briefly summarize the theory behind *d'* and *A'*, and the methods used to evaluate their validity.

**Fig. 1:**
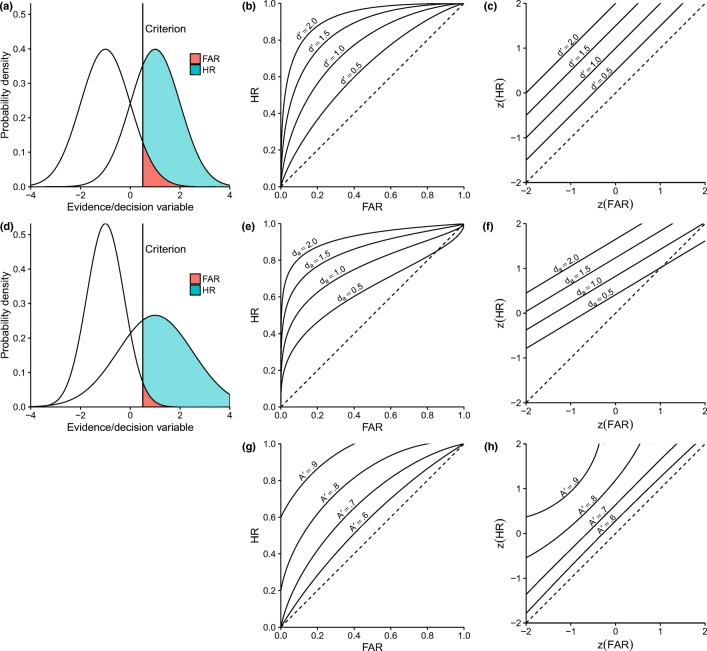
Illustration of noise and signal-plus-noise distribution (first column), receiver operating characteristic (ROC) curves (second column), and ROC curves in *z*-transformed space (*z*ROC; third column) corresponding to *d'* (first row), *d*_*a*_ (second row), and *A'* (third row)

First, *d'* is based on SDT, which, in turn, has its roots in statistical decision theory. For a detailed introduction to SDT, we recommend Green and Swets (1966), Macmillan and Creelman (2005), Wickens (2002), and Gescheider (1997, pp. 105–124). The basic idea of SDT is that when confronted with a binary detection or decision task, cognitive information processing will ultimately result in some type of one-dimensional subjective evidence variable for or against one of the two alternatives (Wickens, 2001, p. 150). This subjective evidence variable is also called the *decision variable* (Macmillan & Creelman, 2005, p. 16). Figure 1a and b show this evidence/decision variable on the *x*-axis. Because the process leading to the evidence is noisy, target-absent (noise) and target-present (signal plus noise) trials both produce a distribution of the decision variable. Whereas the expected value is higher for the target-present trials than for the target-absent trials, the two distributions overlap and do not allow a perfect distinction between the two alternatives. SDT further assumes that individuals derive their decisions by setting a threshold, called the *criterion*, to the decision variable. If the evidence falls short of the criterion, subjects decide that a target is absent (noise); if it exceeds the decision criterion, then they decide that a target is present (signal plus noise). The HR and FAR then each correspond to the cumulative density of one of the two evidence distributions with the criterion as the lower bound (colored areas in Fig. 1a and d). SDT assumes that the criterion can be shifted, with a *liberal* criterion resulting in a higher HR and FAR, and a *conservative* criterion, resulting in a lower HR and FAR. Figure 1a presents an example based on the assumption that the evidence distributions of the two alternatives are normal with equal variance. This equal-variance Gaussian model is the most common model of SDT (Pastore, Crawley, Berens, & Skelly, 2003) and the basis for the detection measure *d'*. In the equal-variance Gaussian model, *d'* is the distance between the means of the two distributions in units of their standard deviation and it fully defines the detection performance, called *sensitivity*. The detection measure *d'* can be calculated as

1 $$ {d}^{\prime }=z(HR)-z(FAR) $$

where z is the inverse of the cumulative distribution function of the standard normal distribution (Green & Swets, 1966). The receiver operating characteristic (ROC) curve (Fig. 1a) describes pairs of HR and FAR values for constant levels of *d'*. If these ROC curves are illustrated in *z* units with *z*(FAR) as the abscissa and *z*(HR) as the ordinate (hereafter, *z*ROC), they form lines with slope 1 and *d'* as their intercept (Fig. 1b).

Whereas SDT is often interpreted as implying the equal variance Gaussian model (Pastore et al., 2003), SDT can also assume other underlying evidence distributions. One example is an SDT model that assumes the two evidence distributions to be normal, but with unequal variance. For a given ratio *s* between the standard deviation of the target-absent (noise) and target-present (signal-plus-noise) distribution, the resulting *z*ROC has slope *s*. For this SDT model, Macmillan and Creelman (2005) proposed using Simpson and Fitter's (1973) detection measure:

2 $$ {d}_a=\sqrt{\frac{2}{1+{s}^2}}\times \left[z(HR)- sz(FAR)\right]. $$

If the ROC curve is known empirically, there are also detection measures that can be estimated without any model assumptions. The most popular of these measures is the area under the curve (AUC; Pepe, Longton, & Janes, 2009). When only one point of the ROC curve is known, Pollack and Norman (1964) provide a *one-point estimation* of the AUC:

3 $$ {A}^{\prime }=\left.0.5+\frac{\left( HR- FAR\right)\left(1+ HR- FAR\right)}{4 HR\left(1- FAR\right)}\ \right|\  HR\ge FAR. $$

By estimating the AUC with one ROC point, *A'* should not be considered assumption-free (Macmillan & Creelman, 2005, p. 103; Wickens, 2001, p. 71). Whereas SDT models make explicit assumptions about the decision process that define the shape of the ROC curves, *A'* also implicitly defines very specific ROC curves as specified by the formula for its calculation. This results in the ROC curves shown in Fig. 1g*.*

To summarize, each one-point detection measure (detection measure based on only one ROC point, i.e., one value for HR and one for FAR), such as *d'* or *A'*, implies a specific ROC curve; that is, a specific assumption about how HR and FAR change when response tendency (i.e., the decision criterion) changes. Whether the implied ROC curve is approximately correct determines whether the detection measure is a valid measure of detection performance. Most importantly, because different detection measures imply different ROC curves, they can lead to different conclusions when, for example, interpreting results of X-ray image inspection tasks.

The shape of the ROC curve for a specific task can be investigated by empirically measuring multiple points of the ROC curve. Macmillan and Creelman (2005) describe four methods with which to gather ROC data from study participants. The first is based on confidence ratings. Instead of providing only a binary decision, the participants provide a rating on a *k*-point Likert scale – for example, ranging from *target certainly absent* to *target certainly present*. Alternatively, the participants deliver the binary response (e.g., *target present* or *target absent*) and then rate their confidence regarding that decision. Each change in level of confidence is then considered as a possible decision criterion (Macmillan & Creelman, 2005, pp. 51–54). With this approach, *k* - 1 ROC points can be derived for *k* response categories.

The other three methods for deriving multiple points of the ROC curve are based on manipulating response tendency (i.e., criterion; Macmillan & Creelman, 2005, p. 71). One method is to manipulate the rewards and costs of a decision (e.g., study participants can be paid according to the amount of hits and false alarms, and the reward of a hit and cost of a false alarm can be manipulated). A second method is to instruct the participants directly to change their criterion by, for example, being conservative in responding *target present* on one set of trials and being more liberal on another set. The third method for gathering ROC points is to manipulate the presentation probability of the signal (Macmillan & Creelman, 2005, p. 72) – the so-called *target prevalence* (Wolfe, Horowitz, & Kenner, 2005). If, for example, most trials contain a prohibited item, subjects will shift their response tendency toward *target present* and therefore achieve a higher HR and FAR. Manipulating the criterion means that each point of the ROC curve requires a separate condition (payoff, instruction, or target prevalence).

Of these four methods, gathering confidence ratings can be applied relatively easily and rapidly, but it is heavily based on the concept of SDT. It is assumed that the subject's decision process is based on a decision variable and that a subject derives a confidence rating from that variable. The other three methods do not require such assumptions because they measure actual decisions under different conditions.

When multiple ROC points are gathered, they can be interpolated to calculate *A*_*g*_ – an estimate of the AUC – without relying on assumptions about the shape of the ROC curve (Pollack & Hsieh, 1969). Hofer and Schwaninger (2004) compared different measures of detection performance and investigated ROC curves derived from confidence ratings in an X-ray image inspection task. They derived ROC curves from pooled confidence ratings and found deviances from symmetrical ROC curves that would be more consistent with the two-state low-threshold theory (Luce, 1963) or non-equal variance Gaussian SDT. However, they also found that *d'*, *A'*, and Δm (a measure for non-equal variance SDT; Wickens, 2001) were highly correlated.

Several other studies using target prevalence manipulations have cast further doubt on the validity of *d'* and *A'* for X-ray baggage inspection. Wolfe et al. (2007) conducted a series of experiments in which subjects performed an X-ray baggage inspection task under varying target prevalence conditions. They found a reduced HR and FAR in low target prevalence conditions with averaged results seeming to lie on a *z*ROC line with a slope of 0.6. Two further publications (Godwin, Menneer, Cave, & Donnelly, 2010a; Van Wert, Horowitz, & Wolfe, 2009) reported *z*ROC slopes similar to those reported by Wolfe et al. (2007), and another study reported a slope of 0.56 (Wolfe & Van Wert, 2010), which is also close to 0.6.

Under Gaussian SDT assumptions, a *z*ROC slope of 0.6 indicates that the target-absent (noise) distribution has a smaller standard deviation than the target-present (signal-plus-noise) distribution. A possible explanation for this is that prohibited items vary in difficulty and this brings additional variation into the target-present distribution.

The aim of our study was to investigate the validity of the detection measures *d'*, *A'*, and *d*_*a*_ and to derive recommendations on how to calculate detection performance in future studies on X-ray image inspection, visual search, and decision tasks. We explored this using two experiments, in which professional X-ray screeners completed a simulated X-ray baggage inspection task. In the first experiment, response tendency (criterion) was manipulated through instruction to test whether it affected the detection measures. The experiment included targets that were known from training and targets that were novel, which resulted in two levels of sensitivity. Valid detection measures should be independent of response tendencies; however, they should differentiate well between different levels of sensitivity. We therefore calculated the effect size of the difference in the detection measures between known and novel targets as an indicator of how well they differentiate between the two levels of sensitivity. In the second experiment, the participants provided confidence ratings that were used to investigate whether the ROC curves are approximately linear in *z*ROC space, as assumed by both *d'* and *d*_*a*_, and to estimate the *z*ROC slope.

## Experiment 1

For this study, we reanalyzed data from Sterchi, Hättenschwiler, Michel, and Schwaninger (2017). The original study evaluated how the rejection rate of screeners can be manipulated, and how performance was related to knowledge about everyday objects. In the experiment, 31 professional screeners completed a simulated X-ray baggage screening task in which the criterion was manipulated directly through instructions. Half of the prohibited items used in the study were known to the screeners from training, whereas the other half were novel. This corresponds to two levels of task difficulty. This experiment allowed us to observe a criterion shift with two levels of sensitivity induced by other means than the previously applied manipulations of target prevalence.

For a detection measure to be valid, it should not be affected by a shift in the decision criterion. In line with the results of the previous studies mentioned above (Godwin, Menneer, Cave, & Donnelly, 2010a; Hofer & Schwaninger, 2004; Van Wert et al., 2009; Wolfe et al., 2007; Wolfe & Van Wert, 2010), we expected the *z*ROC slope to be around 0.6, and therefore for *d'* to decrease when the criterion was shifted to a more liberal level (more target-present responses) in Experiment 1. Both *d'* and *A'* are symmetric – any point (*HR*_*x*_, *FAR*_*x*_) leads to the same value of *d'* and *A'* as (1 − *HR*_*x*_, 1 − *FAR*_*x*_) – and this implies equal variance in terms of SDT (Macmillan & Creelman, 2005, p. 103). We therefore also expected *A'* to decrease when the criterion decreased. As a result of the expected *z*ROC slope of 0.6, a criterion shift should not affect *d*_*a*_ based on that slope. We also aimed at validating *A*_*g*_. As already described in the introduction, *A*_*g*_ is an estimate of the AUC that does not assume a specific shape of the ROC curve but requires multiple ROC points (e.g., derived from confidence ratings) and is therefore not a one-point detection measure like *d'*, *d*_*a*_, or *A'*. Because *A*_*g*_ should not depend on the shape of the ROC curve, it was expected to remain constant. A detection measure should not change when the decision criterion changes; however, it should differentiate well between different levels of ability to detect targets. We therefore analyzed effect sizes of the detection measures when comparing detection performance for the two levels of task difficulty resulting from known and novel prohibited items.

## Method

### Participants

A total of 31 screeners (20 females) from an international airport participated in this experiment. They were all certified screeners, which means that they were qualified, trained, and certified according to the standards set by the appropriate national authority (civil aviation administration) in accordance with the European Regulation (European Commission, 2015). The participating screeners were between 26 and 61 years old (*M =* 45.4, *SD* = 8.9) and had between 2 and 26 years of work experience (*M =* 8.4, *SD* = 5.5). The research complied with the American Psychological Association Code of Ethics and was approved by the Institutional Review Board of the School of Applied Psychology, University of Applied Sciences and Arts, Northwestern Switzerland. Informed consent was obtained from each participant.

### Design

The experiment used a 2 × 2 design with two instructions to manipulate response tendency (normal decision vs. liberal decision) and with two levels of task difficulty (targets known from training vs. novel target items) as within-subject factors. Dependent variables were HR, FAR, *d'*, *d*_*a*_, *A'*, *A*_*g*_, response times, and eye-tracking data.

### Stimuli and materials

The simulated X-ray baggage inspection task contained 128 X-ray images of passenger bags. Of these, 64 images contained one prohibited item (target-present images). They were merged into X-ray images of passenger bags using a validated X-ray image merging algorithm (Mendes, Schwaninger, & Michel, 2011). Four categories of prohibited items were used to create these target-present images: 16 X-ray images contained a gun, 16 images a knife, 16 images an IED, and 16 images contained other prohibited items. To create these 16 X-ray images per threat category, eight threat items per category were each used twice, once in an easy view (as defined by the two X-ray screening experts and the authors) and once rotated (by 85° around the horizontal or vertical axis).

**Fig. 2:**
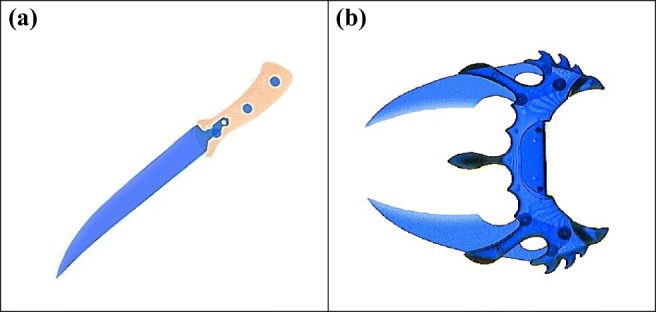
Two examples of the prohibited item category *knife*: (**a**) example of a known target item and (**b**) example of a novel target item (Asian combat knife)

Further, for each threat category, half of the prohibited items were part of the training system (Koller, Hardmeier, Michel, & Schwaninger, 2008; Schwaninger, 2004) used at the particular airport (known targets). The other half of the prohibited items were newly recorded (novel targets). Visual comparisons were used to ensure that they were different from the prohibited items contained in the training system (see Fig. 2 for an example).

All 128 X-ray images were equally divided into four test blocks such that each block contained the same number of known and novel targets per category and viewpoint. X-ray images were presented in a random order within each of the four blocks. The order of the blocks was counterbalanced across the participants.

For eye tracking, we used an SMI RED-m eye tracker with a gaze sample rate of 120 Hz, gaze position accuracy of 0.5°, and spatial resolution of 0.1°. This noninvasive, video-based eye tracker was attached to a 22-in. TFT LCD screen with a resolution of 1,280 × 1,024 pixels placed 50–75 cm from the participant. The stimuli (X-ray images) covered about two-thirds of the screen. Eye tracking was used to examine the users’ eye movements using a post hoc analysis of visual fixations falling within a certain area of interest (AOI). Therefore, in each target-present image, a screening expert manually drew the AOI around the target item (BEGAZE Software; SensoMotoric).

### Procedure

The screeners were tested individually. Each session began with a 9-point calibration of the eye-tracking apparatus. The participants had to follow a moving black dot with their eyes. Then, the task was introduced with on-screen instructions. The screeners were instructed to visually inspect X-ray images of passenger bags by searching for prohibited items and deciding whether each bag was harmless (*target absent*) or might contain a prohibited item (*target present*) and would therefore require a secondary search. The screeners were further instructed that the test contained four blocks. For two blocks, they should inspect (i.e., search and decide) the image as if they were working at a checkpoint (referred to in this article as a *normal decision*). For the other two blocks, they were instructed to visually analyze each object in the X-ray image and decide that the bag was harmless only if each object in the image could be recognized as harmless (*liberal decision*). After the instructions, ten practice trials followed to familiarize the screeners with the task itself and the user-interface of the simulator. The practice trial consisted of five target-absent and five target-present images presented in random order without any feedback on the correctness of the response.

For the test, each trial started with a fixation cross displayed at the center of the screen. After this had been fixated continuously for 1.5 s, it was replaced by an X-ray image. Screeners had to decide whether the content of this image was harmless or not by pressing a key, and then had to give a confidence rating on a 10-point scale ranging from 1 (*very unconfident*) to 10 (*very confident*). There was no feedback on the correctness of responses, and the participants took about 30 min to complete the test.

### Data analysis

A HR of one or FAR of zero leads to an infinite value of *d'* and *d*_*a*_. For the calculation of *d'* and *d*_*a*_, HR and FAR values were therefore transformed using the log-linear rule to correct for extreme proportions (Hautus, 1995), which is one of the two common adjustments to avoid infinite values (Macmillan & Creelman, 2005, p. 8). All within-subject contrasts were tested with exact permutation tests that are appropriate for skewed data and smaller sample sizes. For the estimation of *d*_*a*_, the slope parameter was set to 0.6 in accordance with previous findings from studies that manipulated target prevalence (Godwin, Menneer, Cave, & Donnelly, 2010a; Wolfe et al., 2007; Wolfe & Van Wert, 2010). For *z*ROC slopes and effect sizes, we report bootstrapped BCa-CIs (Efron, 1987) based on 20,000 resamples.

In a review of ROC curves in recognition memory, Yonelinas and Parks (2007) raised the concern that the manipulation of the criterion (i.e., pay-off, instruction, or target prevalence) might also influence sensitivity. In our experiment, we analyzed eye-tracking data to control whether our manipulation also affected search performance and not just decision making. It can be assumed that failure to detect a target can arise from a *scanning error* (Cain, Adamo, & Mitroff, 2013; Kundel, Nodine, & Carmody, 1978; Nodine & Kundel, 1987), where the target is never fixated. If the target is fixated, inspection can still fail because of *recognition* or *decision errors*, and it is unclear whether a distinction between recognition and decision errors is possible and useful (Cain et al., 2013).

In accordance with McCarley's (2009) study, we tested the effect of our manipulation by calculating the proportion of target-present trials with one or more fixations within the AOI (i.e., the location of the target). Rich et al. (2008) also distinguished fixated and non-fixated targets to analyze search errors. They noted that if a target is not fixated, this does not necessarily mean that it was missed during the visual search. However, a target missed during the visual search is more likely to not have been fixated. If the proportion of target-present trials on which the target was fixated is not affected by the manipulation of the criterion, this indicates that the changes in HR and FAR are not caused by search errors in which the study participants simply failed to look at the relevant part of the image (Rich et al., 2008).

## Results

The instructions for the liberal decision condition were designed to change response tendency, that is, to increase the participants' relative frequency of responding with *target present* (rejection rate). A manipulation check revealed an effect of the instruction on the rejection rate with a Cohen's *d* of 0.58. However, ten of the participants did not even show a small increase in the rejection rate (i.e., increase smaller than a Cohen's *d* of 0.20). Because we were interested in whether the detection measures change when participants change their response tendency (and not how successfully we could induce such a change), we excluded participants who did not change their rejection rate from further analysis. The excluded participants did not differ significantly in their HR for known targets (excluded: *M* = .78, included: *M* = .79, *p* = .636), HR for novel targets (excluded: *M* = .63, included: *M* = .58, *p* = .298), or FAR (excluded: *M* = .11, included: *M* = .09, *p* = .570). Table 2 shows the means and standard deviations of the normal decision and liberal decision condition for HR, FAR, *d'*, *d*_*a*_*, A'*, and *A*_*g*_. Exact permutation tests revealed a significantly lower *d'* in the liberal decision condition for both known (*p* = .041) and novel (*p* = .002) targets. Moreover, *A'* was significantly lower for both known (*p* = .034) and novel (*p* = .017) targets. For both *d*_*a*_ (known targets: *p* = .714, novel targets: *p* = .383) and *A*_*g*_ (known targets: *p* = .322, novel targets: *p* = .750), differences did not attain significance. Table 2 also shows the standardized average difference of the detection measures between the two decision conditions as an indicator for the within-subject effect.

**Table 2:** Mean (*SD*) of the normal and liberal decision condition and the effect size (standardized difference) of the decision condition for hit rate (HR), false alarm rate (FAR), and detection measures *d'*, *A'*, *d*_*a*_, and *A*_*g*_

Decision condition	HR	FAR	*d'*	*d*_*a*_	*A'*	*A*_*g*_
	Known targets					
Normal decision	.79 (.10)	.09 (.08)	2.25 (0.61)	2.03 (0.57)	.916 (.044)	.894 (.072)
Liberal decision	.90 (.10)	.25 (.13)	2.01 (0.58)	2.08 (0.61)	.899 (.049)	.906 (.073)
Effect size			-0.40	-0.08	-0.42	0.23
	Novel targets					
Normal decision	.58 (0.14)	.09 (.08)	1.63 (0.41)	1.28 (0.38)	.851 (.040)	.799 (.082)
Liberal decision	.71 (0.13)	.25 (.13)	1.27 (0.44)	1.19 (0.43)	.817 (.074)	.793 (.076)
Effect size			-0.70	-0.19	-0.50	-0.07

The HR and FAR of the two decision conditions were used to calculate individual *z*ROC slopes for known and novel targets separately. The estimated slope had a median of 0.53 (95% BCa-CI [0.24, 0.75]) and a mean of 0.62 (95% BCa-CI [0.34, 1.04]) for known target items, and a median of 0.56 (95% BCa-CI [0.00, 0.83]) and mean of 0.49 (95% BCa-CI [0.27, 0.78]) for novel target items (slopes were first converted into angles of incline and converted back after averaging because steep slopes would otherwise disproportionately influence the mean).

Table 3 summarizes the response time (time from the onset of image display until the submission of the decision by the participant) for correct responses by image type (target-present trials vs. target-absent trials) and decision condition (normal decision vs. liberal decision). For both target-present and target-absent trials, permutation tests indicated a significant difference in response time between normal and liberal decision (target-present trials: *p* = .004, target-absent trials: *p* < .001).

**Table 3:** Response times [ms] for correct responses

	Normal decision		Liberal decision	
	*M* (*SD*)	*Mdn*	*M* (*SD*)	*Mdn*
Target-present	6,000 (2,407)	4,295	8,018 (4,331)	6,291
Target-absent	6,813 (2,798)	5,873	11,162 (6,872)	9,464

*Note.* The reported means and standard deviations are based on individual mean response times, and the reported medians on individual median response times

To control whether the criterion manipulation affected search errors, we calculated the proportion of target-present trials with at least one fixation within the AOI (i.e., the location of the target; see McCarley, 2009). Three participants had to be excluded from the analysis of eye-tracking data because they had either no fixations or no saccades recorded in 73%, 52%, or 24% of their trials, which indicated difficulty with eye tracking for these participants. The remaining 18 participants had a total of 1,151 target-present trials. Twelve (1%) of these had to be excluded because either no fixations or no saccades were recorded. One further trial was excluded because the fixation was in the AOI at the time of stimulus onset. Then, for each participant, the proportion of target images on which the participant fixated the target was calculated separately for the two decision conditions (normal and liberal decision) and the two target types (known and novel targets). Table 4 shows the means and standard deviations of these proportions. The difference between the two decision conditions did not attain significance for either known targets (*p* = .459) or novel targets (*p* = .675), which suggests that the instruction to decide with a more liberal criterion did not affect search errors.

**Table 4:** Mean (*SD*) share of images per subject with a recorded fixation within the area of interest

	Share AOI fixations	
Image type	Normal decision	Liberal decision
Known target	.713 (.237)	.740 (.258)
Novel target	.742 (.165)	.730 (.180)

To investigate the statistical power of the detection measures in terms of reflecting differences in task difficulty (known vs. novel targets) for each detection measure and each of the two decision conditions, we calculated standardized differences (i.e., differences divided by the standard deviation of the differences) as effect sizes of the detection measures between known and novel targets (Table 5). Because *d*_*a*_ is a linear transformation of *d'* when the false alarm rate is constant, the effect sizes of *d'* and *d*_*a*_ were identical.

**Table 5:** Effect size (standardized difference) [and 95% confidence intervals] of target novelty (known vs. novel targets)

	*d'* / *d*_*a*_		*A'*		*A*_*g*_	
Normal decision	1.60	[1.21, 2.10]	1.72	[1.34, 2.15]	1.24	[0.84, 1.64]
Liberal decision	1.98	[1.20, 3.02]	1.73	[1.11, 2.48]	2.20	[1.35, 3.04]

**Fig. 3:**
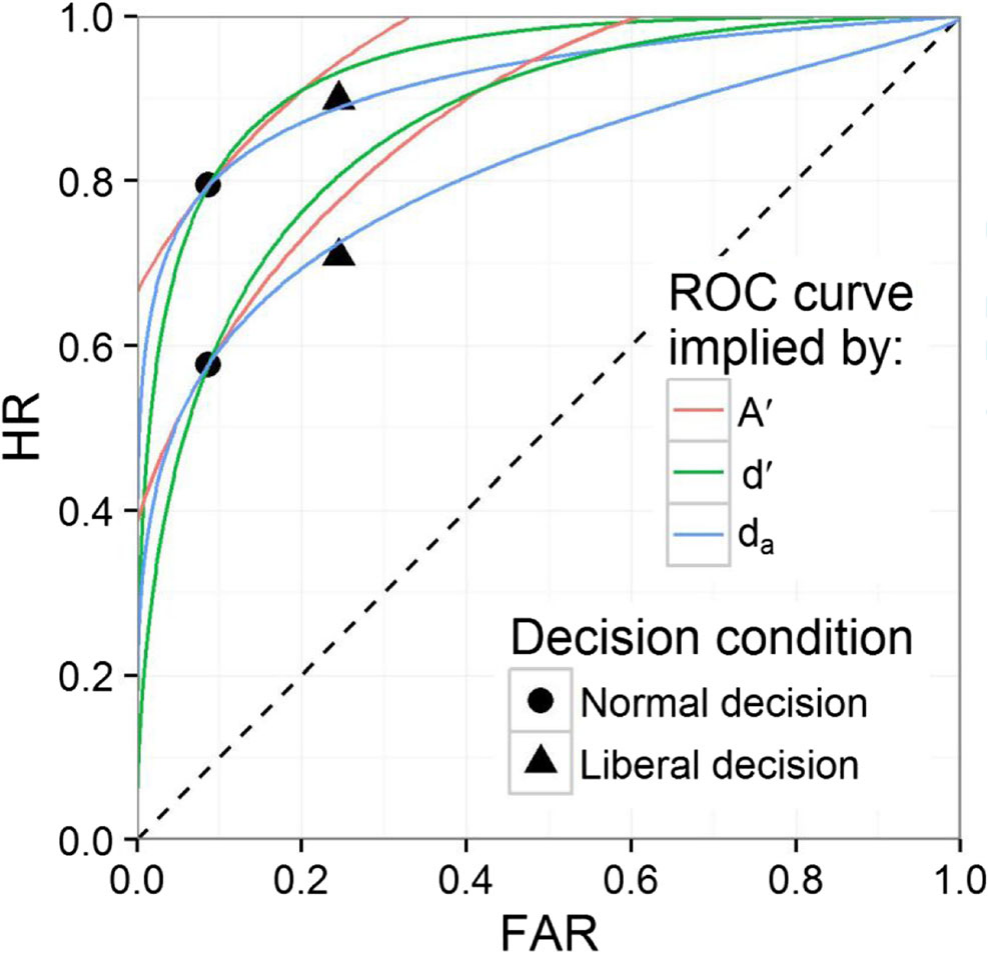
Receiver operating characteristic (ROC) curves implied by *d'*, *A'*, and *d*_*a*_ estimated by the pooled hit rate (HR) and false alarm rate (FAR) of the normal decision condition for known prohibited items (higher HR) and novel prohibited items (lower HR)

Figure 3 shows the ROC curves based on the three detection measures *d'*, *A'*, and *d*_*a*_ of the normal decision condition for known targets (curves with higher HR for a given FAR) and novel targets (curves with lower HR for a given FAR). Because this figure is based on pooled data, it should be interpreted with caution: The aggregation of individual ROC curves can distort their shape, and the figure is therefore not a one-to-one illustration of the tested hypotheses (Yonelinas & Parks, 2007; see the Appendix for a discussion of pooling).

## Discussion

In Experiment 1, we instructed X-ray screeners for one condition to visually inspect X-ray images in the same manner used when they performed their job. For another condition, they were instructed to apply a more liberal decision criterion. Half of the target-present trials contained target items known from training, the other half contained novel target items. As can be seen in Fig. 3, the resulting four points defined by the pooled HR and FAR fit the ROC curve implied by *d*_*a*_ that was set to a slope of 0.6, as suggested by previous research (Godwin, Menneer, Cave, & Donnelly, 2010a; Wolfe et al., 2007; Wolfe & Van Wert, 2010). The permutation tests revealed that *d'* and *A'* values decreased when screeners were instructed to apply a more liberal decision, which casts doubt on the validity of these detection measures in the context of X-ray image inspection. By contrast, *d*_*a*_ with a slope of 0.6 and *A*_*g*_ did not change significantly between the two experimental conditions.

The fact that the instructed, more liberal criterion caused a decrease in *d'* and *A'* is in line with previous findings of changes in *d'* when target prevalence manipulations induced a shift in the criterion (Godwin, Menneer, Cave, & Donnelly, 2010a; Wolfe et al., 2007; Wolfe & Van Wert, 2010). The results of these studies also suggest that *d'* and *A'* can lead to wrong conclusions when used to decompose a unidirectional change of HR and FAR into sensitivity and criterion changes.

When trying to induce a criterion shift using experimental manipulation, there is a risk that the manipulation might also affect sensitivity (Yonelinas & Parks, 2007). In our experiment, the given instruction to decide more liberally slowed the response times. Similarly, studies that manipulated target prevalence also found slower responses in high target prevalence conditions (Godwin, Menneer, Cave, & Donnelly, 2010a; Wolfe et al., 2007; Wolfe & Van Wert, 2010). Our main findings should be robust regarding a potential change in sensitivity for two reasons: First, we found no difference in the share of images with target fixation between the two decision conditions. This supports the assumption that the observed change in HR and FAR was caused by a change in decision making and not a change in search errors (McCarley, 2009; Rich et al., 2008). Second, if the manipulation affected sensitivity, then one would expect higher sensitivity in the liberal decision condition in which response times were longer (following the line of argument in Wolfe et al., 2007). Such an accidental effect on sensitivity could therefore not explain the decrease we found in *d'* and *A'*.

## Experiment 2

In Experiment 1, we calculated *d', A'*, and *d*_*a*_*,* for which we set the slope to 0.6 based on previous findings (Godwin, Menneer, Cave, & Donnelly, 2010a; Wolfe et al., 2007; Wolfe & Van Wert, 2010). *d*_*a*_ was found to be a more valid detection measure than *d'* and *A'*. However, estimations of the slope parameter with the data from Experiment 1 resulted in large confidence intervals. Further, ten of the participants were excluded because they failed the manipulation check, which might have biased the sample. Experiment 2 was therefore intended to provide a more precise estimation of the slope parameter and to further investigate the validity of detection measures using another methodological approach: multiple ROC points were obtained by analyzing confidence ratings. In comparison to Experiment 1, the criterion was not manipulated directly, and the test therefore included more trials per participant and condition.

## Methods

### Participants

A total of 124 professional, certified cabin baggage screeners (68 female) from an international airport participated in Experiment 2. The participants were between 22 and 64 years old (*M* = 44.3, *SD* = 11.2; one participant did not report his/her age) and they had up to 29 years of work experience (*M* = 7.1, *SD* = 5.6; seven participants did not report their work experience). The research complied with the American Psychological Association Code of Ethics and was approved by the Institutional Review Board of the School of Applied Psychology of the University of Applied Sciences and Arts, Northwestern Switzerland. Informed consent was obtained from each participant.

### Stimuli and materials

The test consisted of 128 X-ray images of real passenger bags. Half of these images contained a prohibited item. The merging of the prohibited items into the bag images was performed in the same manner as in Experiment 1 using a validated algorithm (Mendes et al., 2011). Four categories of prohibited items were used: 16 images contained a gun, 16 images a knife, 16 images an IED, and 16 explosive material. Each prohibited item appeared twice, once in an easy view and once rotated. None of the prohibited items were part of the training system used at the particular airport. The 128 images were equally divided into two blocks with each block containing the same number of targets per category and view. Images were presented in a random order within the block. The order of the two blocks was counterbalanced across the participants.

### Procedure

The participants were tested in groups of maximally six screeners at a time. The screeners had to inspect the X-ray images for prohibited items. If they detected a prohibited item, they had to mark its location in the image (this was conducted for another study). They had to press a key to decide whether the bag was harmless or not, and they then had to assign a confidence rating on a 5-point scale ranging from 1 (*very unconfident*) to 5 (*very confident*). To become familiar with the test, the instruction was followed by eight practice trials, on which the screeners received feedback on the correctness of the responses. During the test itself they did not receive feedback. Participants were allowed to take a short break after the first half of the test that lasted for 1 min in average. Participants took about 20 min to complete the test.

### Data analysis

For each participant, the HR and FAR were calculated for the different levels of confidence rating according to Macmillan and Creelman (2005, pp. 51–54), resulting in nine ROC points per participant.

To estimate individual slope parameters based on the confidence ratings, we used the maximum likelihood estimation algorithm LABROC4 developed by Metz, Herman, and Shen (1998). Because the slope parameter is the ratio of two differences in two variables, it is inappropriate to directly calculate its mean (because steep slopes result in large numbers, a horizontal *z*ROC, for example, has a slope of zero and a vertical *z*ROC has a slope of infinity and the mean of the two slopes would only consider the vertical slope). We therefore arctan-transformed the slope parameters into angles of incline before averaging, and then transformed them back for interpretability.

### Results

One participant provided the maximum confidence level for all trials and was therefore excluded. A second participant had to be excluded because all derived ROC points for FAR were either zero or one, not allowing for a maximum likelihood estimation of the slope parameter. The remaining 122 participants achieved a mean HR of .70 (*SD* = .07) with a mean FAR of .07 (*SD* = .05). The response time (time from the onset of the image display until the submission of the decision by the participant) is summarized in Table 6 for correct responses by image type (target-present trials vs. target-absent trials).

**Table 6:** Response times [ms] for correct responses

	*M* (*SD*)	*Mdn*
Target-present	4,781 (1,087)	3,816
Target-absent	5,079 (1,959)	4,008

*Note.* The reported group means and standard deviations are based on individual mean response times, and the reported medians on individual median response times

**Fig. 4:**
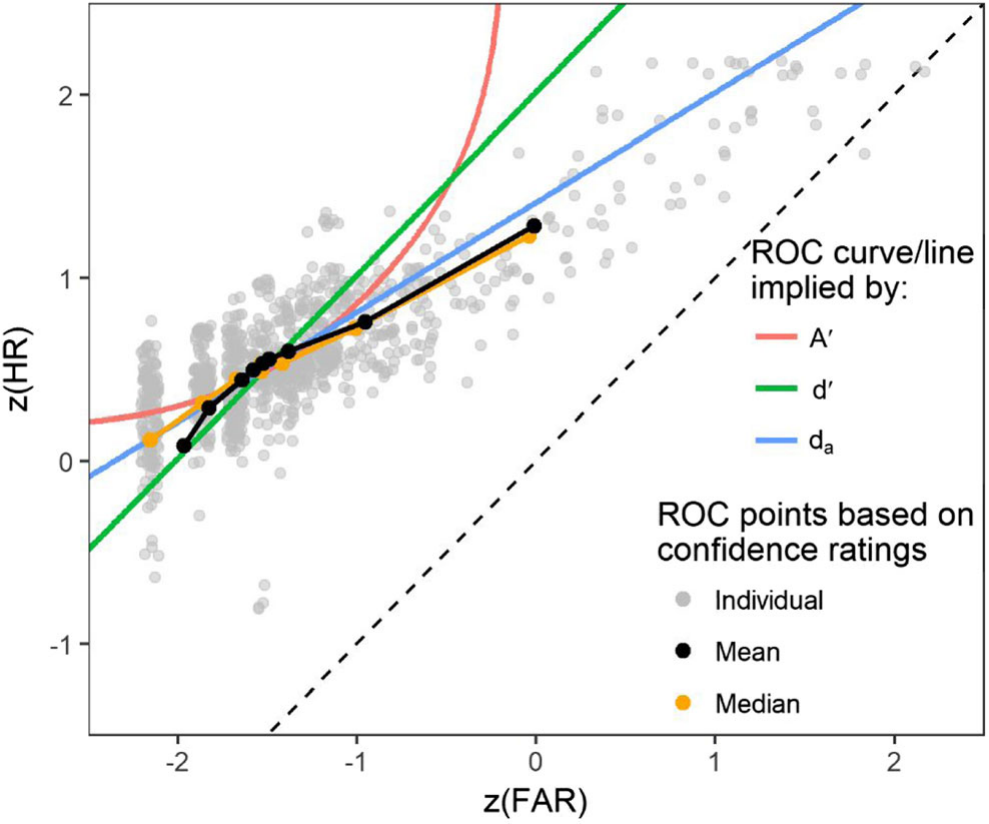
Individual (grey; jittered) and pooled (black) empirical *z*ROC curves, the lines corresponding to the mean *A'*, *d'*, and *d*_*a*_ with a slope of 0.6, and the chance line (dashed)

Figure 4 shows individual *z*ROC points and the averaged *z*ROC curves based on confidence ratings (for a discussion of pooling ROC curves see the Appendix). The averaged *z*ROC curves seem to better fit the *z*ROC curve predicted by *d*_*a*_ based on a slope of 0.6 than those predicted by *d'* or *A'* (one exception is the mean of the leftmost *z*ROC point, which, however, is distorted downwards as a result of the necessary exclusion of ROC points with a false alarm of zero that are not defined in *z*ROC space).

**Fig. 5:**
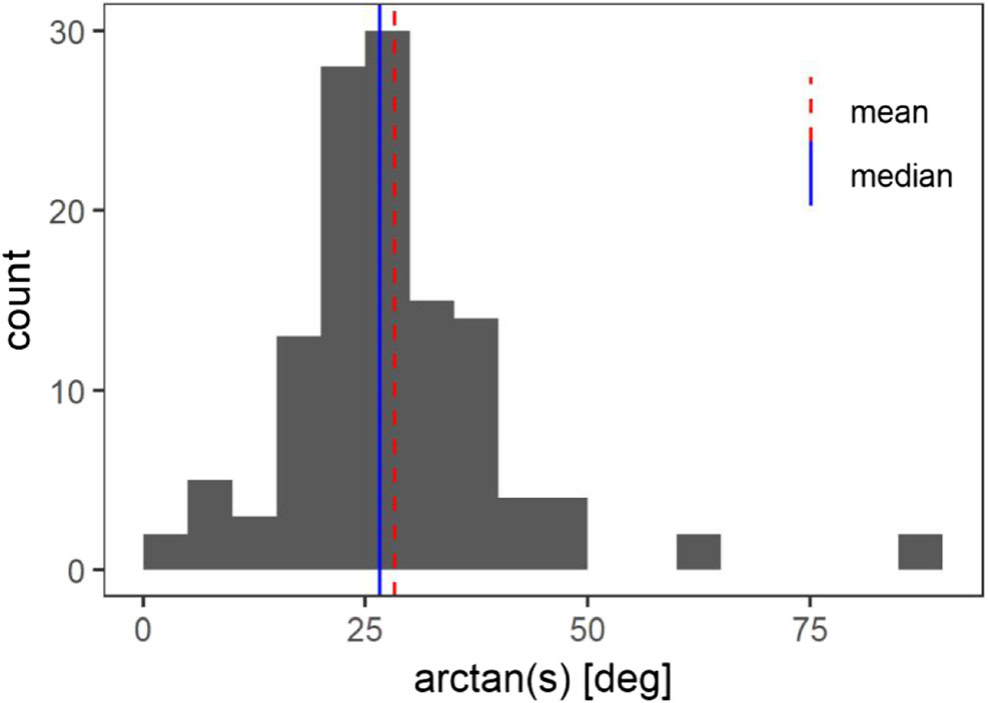
Distribution, mean (red dashed line), and median (solid blue line) of arctan-transformed individual slope parameters

Arctan-transformed individual slope parameters (i.e., angles of incline) estimated using the LABROC3 algorithm (Metz et al., 1998) are illustrated in Fig. 5. When transformed back, they show a mean of 0.54 (95% BCa-CI [0.50, 0.60]) and median of 0.50 (95% BCa-CI [0.46, 0.55]).

## Discussion

In Experiment 2, the participants completed an X-ray baggage inspection task providing confidence ratings for each image. The pooled *z*ROC points and the estimated *z*ROC slopes of around 0.5–0.6 confirm the findings of Experiment 1 that *d'* and *A'* overestimate HR, or underestimate FAR when the criterion is shifted and becomes more liberal. The pooled *z*ROC curves were approximately linear, which supports the validity of *d*_*a*_ for the X-ray baggage inspection task in line with the results of Wolfe and Van Wert (2010). The results show a mean slope of 0.54, close to other studies that reported *z*ROC slopes of around 0.6 (Godwin, Menneer, Cave, & Donnelly, 2010a; Wolfe et al., 2007) and another study that reported a slope of 0.56 (Wolfe & Van Wert, 2010).

Despite the similar *z*ROC slopes found in these studies, one should be cautious to always adopt *d*_*a*_ with a slope of 0.5–0.6 for any X-ray baggage inspection or other visual search task. A non-unit slope *z*ROC implies that there is a point at which the ROC curve falls below the chance line, where the FAR exceeds the HR (Macmillan & Creelman, 2005, p. 68). When sensitivity is sufficiently high, this becomes negligible because it only concerns values very close to the limits of the ROC space. However, for low sensitivity (e.g., for difficult items or inexperienced X-ray screeners), a *z*ROC with a slope of 0.5–0.6 implies below-chance performance for a possibly relevant range of the decision criterion (see Fig. 1e). It would therefore be reasonable to assume that the *z*ROC slope converges to a unit slope with decreasing sensitivity. Such a convergence has been found repeatedly in research on recognition memory (Brown & Heathcote, 2003; Glanzer, Kim, Hilford, & Adams, 1999; Hirshman & Hostetter, 2000; Ratcliff, McKoon, & Tindall, 1994).

In addition to the level of sensitivity, other factors might influence the slope parameter. There is some empirical evidence that the *z*ROC slope might vary between different implementations of the X-ray baggage inspection tasks or depending on the participants: Alongside our findings and other studies reporting *z*ROC slopes around 0.5–0.6 (Godwin, Menneer, Cave, & Donnelly, 2010a; Wolfe et al., 2007; Wolfe & Van Wert, 2010), one study found a lower *d'* for lower target prevalence (Wolfe, Brunelli, Rubinstein, & Horowitz, 2013), which indicates a *z*ROC slope larger than one. There are also a few studies that show an effect of target prevalence on HR and FAR without a significant effect on *d'* (Godwin, Menneer, Cave, Helman, et al., 2010b; Ishibashi et al., 2012) or *A'* (Godwin, Menneer, Cave, Thaibsyah, & Donnelly, 2015). They therefore do not contradict a unit-slope *z*ROC. To summarize, whereas it is reasonable to infer that a *z*ROC slope is around 0.5–0.6 for many visual inspection, visual search, and decision tasks with X-ray images, this might not be always true. In the following section we discuss how this issue can be addressed in future studies.

## General discussion

To investigate the validity of two detection measures commonly used in visual search and decision tasks such as airport security and medical screening, we conducted two studies with different methodological approaches. Experiment 1 manipulated the criterion by direct instruction, whereas Experiment 2 used confidence ratings to generate multiple ROC points. For both studies, *d'* and *A'* were found to be invalid detection measures for the investigated X-ray baggage inspection tasks. More specifically, *d'* and *A'* would have wrongly indicated lower sensitivity for a more liberal decision criterion.

Studies investigating the effect of target prevalence on X-ray baggage inspection tasks also found *d'* to indicate lower sensitivity for more liberal decision criteria where equal or lower sensitivity would be expected (Godwin, Menneer, Cave, & Donnelly, 2010a; Wolfe et al., 2007; Wolfe & Van Wert, 2010). Our studies extend this research by showing that this phenomenon is not specific to the effect of target prevalence but also holds for other means of manipulating the criterion, and therefore seems to be a property of the ROC curve of the X-ray baggage inspection task in general.

Despite *A'* not making any assumptions about the underlying decision processes, *A'* implies a very specific and symmetric ROC curve (Macmillan & Creelman, 2005). It should therefore not be expected to have an advantage over *d'*, which the results of our studies confirmed. The general discussion and our recommendations will therefore focus on *d'* and *d*_*a*_.

When lifting the assumption of equal variance, the Gaussian SDT model is extended by an additional parameter: the ratio *s* between the standard deviation of the signal-plus-noise (target-present) and noise (target-absent) distribution. The Gaussian SDT model assumes an ROC curve that becomes a straight line when *z*-transformed with parameter *s* as its slope. For detection measure *d*_*a*_, which corresponds to this model, to be valid for X-ray baggage inspection tasks, *z*ROC curves should be approximately linear. In line with a study from Wolfe and Van Wert (2010), the results of Experiment 2 show approximately linear pooled *z*ROC curves. In our experiments, the slope parameter was around 0.5–0.6, which corresponds well with the findings in other experiments that investigated the X-ray baggage inspection task (Godwin, Menneer, Cave, & Donnelly, 2010a; Wolfe et al., 2007; Wolfe & Van Wert, 2010). However, the slope parameter might depend on the level of sensitivity and might vary between different implementations of the X-ray baggage inspection tasks or depending on the participants.

To better understand what factors influence the slope parameter, a better understanding of the inspection process would be useful and should be the focus of future studies. From the perspective of Gaussian SDT, a *z*ROC slope smaller than one implies that the signal-plus-noise distribution has a higher standard deviation than the noise distribution. A possible explanation for this is that prohibited items can vary strongly in how well they can be recognized – for example, depending on item category (Halbherr et al., 2013; Koller et al., 2009) and the exemplar within categories (Bolfing, Halbherr, & Schwaninger, 2008; Schwaninger et al., 2007). The SDT framework might have to be extended to provide a better model of the visual inspection process. For instance, Wolfe and Van Wert (2010) described the task as successive decisions for single items within the X-ray image. This model assumes that the observer makes a decision according to SDT for one item after the other until the observer either decides that an item is prohibited or a quitting threshold is reached. Conceptually, this is similar to the two-component model of visual inspection by Spitz and Drury (1978), which has been applied to the visual inspection of X-ray images and consists of visual search and decision processes (Koller et al., 2009; Wales et al., 2009). For modeling recognition memory, SDT has been extended in various forms by assuming that recognition can be based on either recollection or familiarity (Yonelinas & Parks, 2007). Similarly, different types of recognition might apply in X-ray baggage inspection – some items might be recognized with certainty, whereas for other items, a decision has to be made under high uncertainty.

Our studies and the reviewed literature focus on the task of inspecting X-ray images of passengers’ cabin baggage. Our findings do not necessarily directly translate to related domains, such as the inspection of medical X-ray images or other visual search tasks with artificial stimuli; however, such related domains should also not expect *d'* and *A'* to be valid without further consideration. Future research should specifically investigate to what extent the findings we report also apply in related domains.

We hope that future research will provide more insights into the image inspection process; however, we suggest a critical yet pragmatic approach when investigating performance in image inspection tasks. As famously stated by Box (Box & Draper, 1987, p. 424), “all models are wrong, but some are useful.” In X-ray image inspection, the main use of a detection measure is to identify whether a unidirectional difference in HR and FAR (i.e., when both HR and FAR are higher in one group or condition) is only a difference in the decision criterion or also a difference in detection performance in terms of sensitivity. That is, a comparison of detection measures should answer the question of who would have the higher HR and lower FAR if everyone used a similar decision criterion.^1^ For one-point detection measures, the implied ROC curve therefore needs to be approximately correct. Our studies and the reviewed literature show that for X-ray baggage inspection, this is often not the case for *d'* and *A'*. Instead, *d*_*a*_ with a *z*ROC slope of 0.5 to 0.6 often seems to provide the better measure. However, while it is not clear what factors determine the *z*ROC slope, we recommend testing *d*_*a*_ with a slope of 0.5 in addition to *d*_*a*_ with a slope of 1 (i.e., *d'*) as the upper and lower bound, respectively. Another approach is to gather confidence ratings and use *A*_*g*_ as a detection measure. Whereas *d'*, *A'*, and *d*_*a*_ imply a specific shape of ROC curve, *A*_*g*_ is conceptually valid for any form of ROC curve. However, it requires the collection of confidence ratings, and is based on the assumption that these confidence ratings allow a prediction of alternative criterion locations at an individual level. Moreover, some methodological problems can arise because *A*_*g*_ estimates the AUC by linearly interpolating empirical ROC points (Pollack & Hsieh, 1969). This approach increasingly underestimates the AUC with a decreasing number of ROC points (Macmillan & Creelman, 2005, p. 64). *A*_*g*_ might therefore require a relatively high number of trials to be a valid detection measure. In Experiment 1, *A*_*g*_ performed acceptably well – it was not significantly affected by the manipulation of the decision condition, and differentiated between known and novel targets with statistical power comparable to *d*_*a*_. However, this is only limited support for the measure, as the results are restricted to a within-subject comparison of a small sample. Future research might clarify whether confidence ratings allow a reliable prediction of criterion shifts induced by changes in target prevalence or instruction.

In conclusion, X-ray image inspection research and related domains will have to be cautious when using one-point estimates of sensitivity such as *d'* and *A'*. We recommend always starting by performing an analysis and discussion of the directly accessible HR and FAR. Estimating the sensitivity and criterion is often only necessary if HR and FAR are affected unidirectionally. In that case, it should be considered that a *z*ROC slope can be expected to lie somewhere between 0.5 and 1 for X-ray baggage inspection tasks. With *d*_*a*_, effects on sensitivity can be estimated for these two slopes separately to test the two limits of the assumption of constant sensitivity (where the upper limit with a *z*ROC slope of 1 corresponds to *d'*). Collecting confidence ratings allows to directly estimate the *z*ROC slope for the investigated task, to calculate *A*_*g*_, which provides an additional estimation of sensitivity, and help to further understand the shape of the ROC curve in X-ray image inspection.

## Figures and Tables

**Fig. 6:**
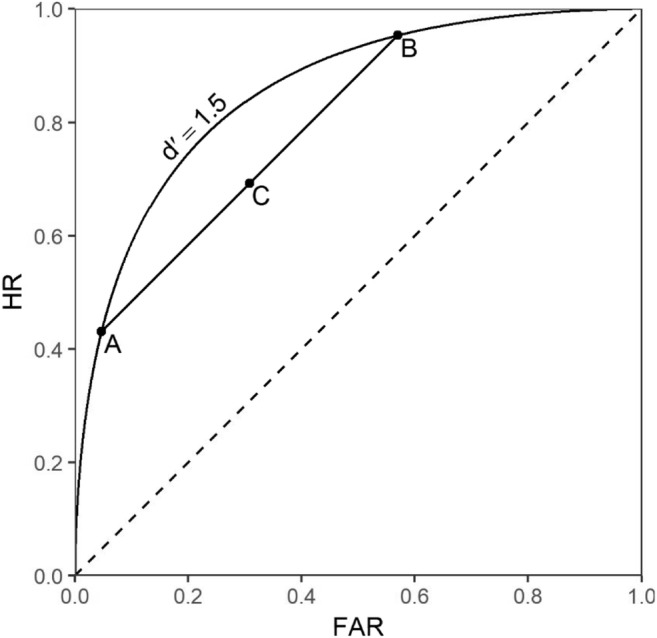
When the two points A and B from the same receiver operating characteristic (ROC) curve are averaged, the resulting ROC point C is below the original ROC curve

**Fig. 7:**
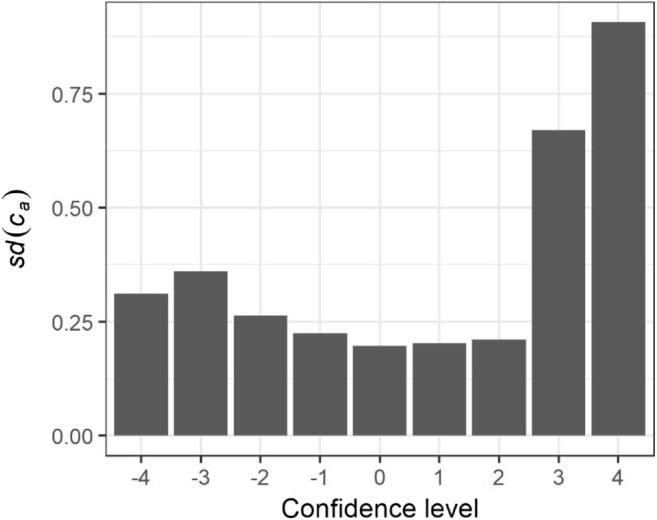
Between-subject standard deviation of c_a_ (based on a slope of 0.6) for each confidence level

**Fig. 8:**
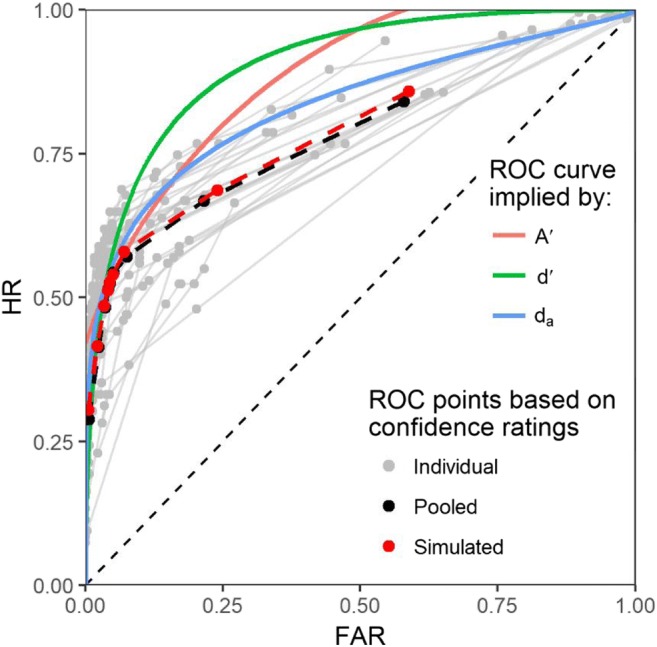
Receiver operating characteristic (ROC) points based on individual (gray) and pooled confidence rating data of dataset 2 (black, dashed), created from a simulation (red, dashed), as assumed by the average *d'* (green), *d*_*a*_ (blue), and *A'* (red)

## Appendix

### Pooling and ROC curves

When investigating receiver operating characteristic (ROC) curves based on the framework of signal detection theory (SDT), in almost all experiments of real interest, some type of averaging must be performed (Macmillan & Creelman, 2005, p. 331). For X-ray image inspection, combining different stimuli in an experiment seems reasonable because this is representative of this task in the real world. However, when responses from different subjects are averaged, the resulting ROC curve can deviate systematically from individual ROC curves, as we will illustrate in the following paragraphs.

Figure 6 assumes two subjects with an identical ROC curve in the shape assumed by Gaussian SDT. If these subjects differ in their decision criterion, their averaged ROC point (i.e., hit and false alarm rate) will lie in the middle of the line connecting their individual ROC points and therefore below their true ROC curve. How far away the averaged ROC point is from the true ROC curve depends on the difference between the decision criteria (i.e., the distance between the individual ROC points) and on the curvature of the ROC. When looking at pooled ROC points, it is therefore important to consider the between-subject variation in decision criteria. Plotting ROC curves based on confidence ratings now assumes that each level of the confidence rating could be a possible criterion and therefore each confidence level provides an ROC point (one of them is guaranteed to be at a HR and FAR of one, therefore *k* confidence levels result in *k*-1 meaningful ROC points). Figure 7 shows that for Experiment 2, the variation between the individual criteria is different between the confidence levels. Some of the ROC points based on pooled data should therefore be further away from the "true" ROC curve.

Figure 8 shows individual and pooled ROC points of Experiment 2 in comparison with the theoretical ROC curves based on the average *d'*, *d*_*a*_, and *A'*. As expected, particularly the two most liberal (i.e., rightmost) ROC points fall below the theoretical ROC curves.

To test whether the deviation from the theoretical ROC curves could be the mere result of pooling, we ran a simulation. The simulation assumed that the ROC curve based on *d*_*a*_ with a slope parameter of 0.6 holds true for each individual and, for simplification, that individuals deviate normally from the mean *d*_*a*_ of Experiment 2 (*M* = 1.37) with the standard deviation of Experiment 2 (*SD* = 0.26). Additionally, for the criterion *c*_*a*_ of each confidence level, it was assumed that subjects vary normally around the group's average, and again, these parameters were estimated using Experiment 2. According to these assumptions 10,000 observations were created for each confidence level and pooled. The result of this quite simple simulation is also depicted in Figure 8 and falls close to the pooled ROC points from the original data. This suggests that the pooled ROC points might simply deviate from the ROC curve based on *d*_*a*_ because of the variation in the criterion and sensitivity between subjects (however, this does not, of course, prove that the pooled ROC curve would look like the ROC curve based on *d*_*a*_ if all pooling artifacts were eliminated).

As illustrated, pooling ROC points can severely distort the shape of ROC curves. The illustrated problems of pooling should not occur if averaging is performed after *z*-transformation and the *z*ROC curves are linear. However, *z*-transformation before pooling is often not fully possible because of FAR or HR values of zero or one on an individual level, for which the *z*-transformation (i.e., the inverse of the cumulative distribution function of the standard normal distribution) is undefined.

## Footnotes

1 For different levels of sensitivity, it is conceptually not clear what constitutes an equal decision criterion (Macmillan & Creelman, 2005, pp. 36–44).
